# Challenges in drug discovery targeting TriTryp diseases with an emphasis on leishmaniasis

**DOI:** 10.1016/j.ijpddr.2018.09.006

**Published:** 2018-09-28

**Authors:** Laura M. Alcântara, Thalita C.S. Ferreira, Fernanda R. Gadelha, Danilo C. Miguel

**Affiliations:** Biology Institute, University of Campinas – UNICAMP, Campinas, São Paulo, Brazil

**Keywords:** Chemotherapy, Drug development, *Leishmania*, Public-private partnership, Trypanosomatids

## Abstract

Tritryps diseases are devastating parasitic neglected infections caused by *Leishmania* spp., *Trypanosoma cruzi* and *Trypanosoma brucei* subspecies. Together, these parasites affect more than 30 million people worldwide and cause high mortality and morbidity. Leishmaniasis comprises a complex group of diseases with clinical manifestation ranging from cutaneous lesions to systemic visceral damage. Antimonials, the first-choice drugs used to treat leishmaniasis, lead to high toxicity and carry significant contraindications limiting its use. Drug-resistant parasite strains are also a matter for increasing concern, especially in areas with very limited resources. The current scenario calls for novel and/or improvement of existing therapeutics as key research priorities in the field. Although several studies have shown advances in drug discovery towards leishmaniasis in recent years, key knowledge gaps in drug discovery pipelines still need to be addressed. In this review we discuss not only scientific and non-scientific bottlenecks in drug development, but also the central role of public-private partnerships for a successful campaign for novel treatment options against this devastating disease.

## Highlights

• Treatment options targeting TriTryp diseases are limited.
• Scientific and non-scientific bottlenecks need to be unveiled for the development of new treatments.
• Private and public sector partnership is key to allow advances in bench-to-bedside science.

## Graphical abstract

**Figure:**
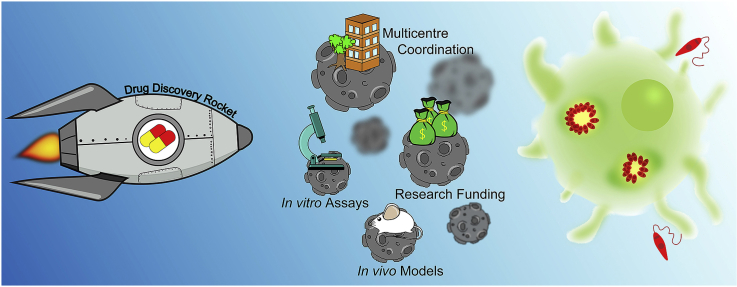

## 1 Background

*Leishmania* spp., *Trypanosoma cruzi* and *Trypanosoma brucei* subspecies are the causative agents of leishmaniasis, American trypanosomiasis (Chagas disease) and Human African trypanosomiasis (sleeping sickness), respectively. Together, these protozoal infections are known as TriTryp diseases. They represent a serious public health problem worldwide, especially in Africa, South America and Asia. TriTryp diseases are responsible for high mortality and morbidity rates in developing countries and impact affected regions economically and socially (Barrett et al., 2003; Hotez et al., 2009; WHO, 2018a). As there are no vaccines available, the treatment of infected people is one of the main strategies to control these diseases. However, drugs in use present major drawbacks, such as high toxicity, relevant contraindications and complicated administration regimens (Table 1) (Nussbaum et al., 2010; Singh et al., 2012).

**Table 1:** TriTryp diseases.

	Leishmaniasis	American Trypanosomiasis (Chagas disease)	Human African Trypanosomiasis (sleeping sickness)
Causative agent	*Leishmania* species (*Leishmania* and *Viannia* subgenera)	*Trypanosoma cruzi*	*Trypanosoma brucei* subspecies
Endemic region	Mainly in Asia, South America, East Africa, and Mediterranean countries	Mainly in Latin America	Exclusively in Africa
Clinical manifestation	Cutaneous Leishmaniasis (skin lesions and mucous ulcers) Visceral Leishmaniasis (enlarged spleen and liver, fever, pallor)	Acute phase with variable symptoms (fever, headache, enlarged spleen and liver) Chronic infections: cardiac and/or digestive forms (megaesophagus and megacolon)	General manifestations: fever, headaches, neurological manifestation: seizures, poor coordination, somnolence, coma
Current treatments	Pentavalent antimonials, Amphotericin B, miltefosine and paromomycin	Benznidazole and nifurtimox	Suramin, pentamidine, melarsoprol, eflornithine, and nifurtimox-eflornithine combination
Disadvantages of chemotherapy	Toxicity, severe side effects, hospitalization requirement and parasite resistance emergence	Variable response in chronic disease, poor tolerability, severe toxic effect and contraindications	High toxicity and inefficacy against the neurologic phase

## 2 *Leishmania* and leishmaniasis

Leishmaniasis is a complex group of diseases caused by different species of protozoan parasites that are members of the genus *Leishmania*, and impose a serious public health problem worldwide. According to the World Health Organization (WHO), leishmaniasis is endemic in 98 countries affecting around 12 million people. It is estimated that over 1 billion people live in endemic areas at risk of infection. Also, around 1.3 million new cases of the disease are registered annually and death counts 20,000 to 30,000 per year (Alvar et al., 2012; WHO, 2018a).

*Leishmania* has a digenetic life cycle, involving both invertebrate (phlebotominae sandflies) and vertebrate (mammals, including humans) hosts and presents two very distinct stages: promastigotes (extracellular and flagellated forms found in the insect gut) and amastigotes (intracellular and round forms that multiply within phagocytic immune cells). Mammals are infected by the bite of female sandflies that regurgitate infective promastigotes during a blood meal. Upon host infection, promastigotes are phagocytosed mainly by macrophages, where they differentiate into amastigotes inside phagolysosomal compartments. After successive multiplication, amastigotes are released from macrophages and re-infect new cells, such as macrophages, dendritic cells and fibroblasts. Occasionally, sandflies become infected by ingesting infected cells during next blood meal (Killick-Kendrick, 1990; Sacks and Kamhawi, 2001).

The disease leads to different clinical manifestations determined both by host parameters, such as genetic characteristics and immunological status (Jeronimo et al., 2007; Blackwell et al., 2009; Sakthianandeswaren et al., 2009), and parasite features, including heterogeneity in the virulence of different species/strains (Naderer et al., 2004). Clinical manifestations range from cutaneous lesions (cutaneous leishmaniasis, CL) and mucous ulcers (mucocutaneous leishmaniasis, MCL) to systemic visceral damage (visceral leishmaniasis, VL). VL is the most severe form of the disease and is potentially fatal if untreated (Piscopo and Mallia Azzopardi, 2007). Bangladesh, Brazil, India, Ethiopia, Kenya, Nepal and Sudan concentrate more than 90% of world's VL cases, while CL and MCL are predominantly diagnosed in Afghanistan, Algeria, Colombia, Brazil, Iran and additional African and Latin countries (Alvar et al., 2012; WHO, 2018a).

Currently, chemotherapeutic options show major disadvantages limiting the treatment of infection and clinical success (Table 1). Pentavalent antimonials (Glucantime^®^ and Pentostam^®^), Amphotericin B (Fungizone^®^ – salt formulation and Ambisome^®^ - liposomal formulation), miltefosine (Impavido™) and paromomycin (Humatin^®^) are classically used for the treatment of leishmaniasis; however, these drugs present a number of limitations, including high cost, limited efficacy, and disabling side effects due to high toxicity and extended period of treatment. Of all the above drugs, miltefosine is the only one administered orally. Also, the emergence of antimonial-resistant *Leishmania* strains and variable susceptibility regarding distinct species/strains have been reported (Croft et al., 2006a; Barrett and Croft, 2012; Freitas-Junior et al., 2012; Uliana et al., 2017). Collectively, these factors contribute to the therapeutic failure observed in clinical practice.

Given the epidemiologic impact of leishmaniasis as well as the lack of appropriate treatment options, the development of safer, more effective and affordable new drug candidates and/or the improvement of existing therapies remains a priority.

## 3 Drug discovery criteria regarding leishmaniasis

**Fig. 1:**
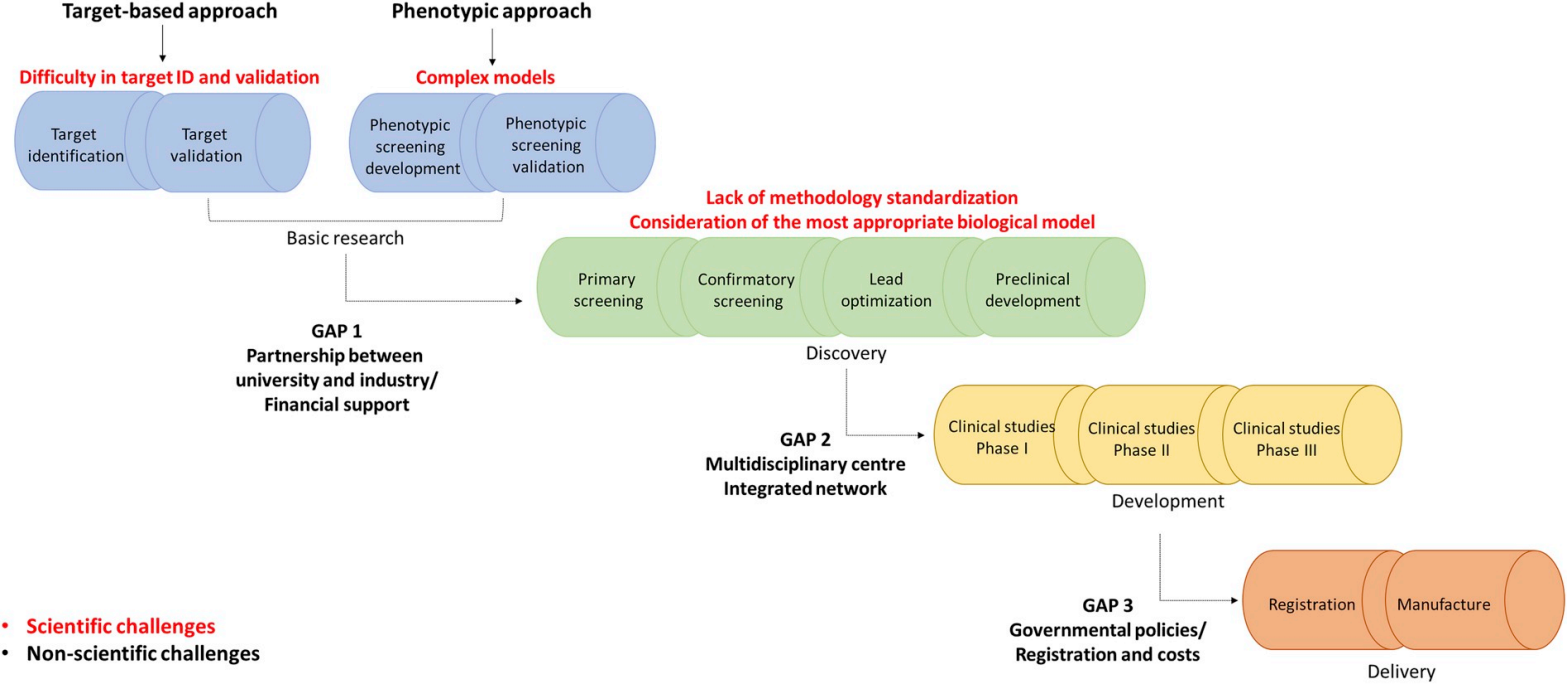
**Classical pipeline for drug discovery highlighting scientific and non-scientific challenges.** The process often starts with basic research in order to (i) identify and validate molecular/biochemical targets (target-based assays) or (ii) develop and validate phenotypic assays (cell-based assays), in which compounds are tested against the whole parasite or a given biological system. Medicinal chemistry experts will then optimize selected compounds (*hits*). Next steps consist in testing candidates in animal models and assessing their performances by determining pharmacokinetics and pharmacodynamics properties. Finally, a compound is targeted to clinical trials in humans and, once showing a satisfactory profile, it is defined as a drug candidate. The last steps of the pipeline include registration and manufacture of the medicine.

Despite the advances observed in the anti-*Leishmania* drug discovery field, the innovation cycle is a challenging process that still faces gaps (Fig. 1).

Several approaches have been reported to identify and optimize new candidates against *Leishmania* parasites, including *de novo* drug discovery (Fig. 1), focusing on the identification of new chemical entities by screening both chemical and natural product libraries (Siqueira-Neto et al., 2012; Annang et al., 2015; Khare et al., 2016; Peña et al., 2015; Zulfiqar et al., 2017), and *short-term* strategies, including combinatory therapies, new formulations for drugs in use and drug repurposing (Alirol et al., 2013; Andrews et al., 2014; Hamill, 2013; Trinconi et al., 2014).

Target Product Profile (TPP) - defined as a planning tool for promising therapeutic candidates - has a major role in *de novo* drug discovery. Basically, TPP takes into account factors, such as compounds’ clinical efficacy, delivery mode, safety and tolerability, contraindications, dosage form, stability, treatment duration, and cost (Bandyopadhyay, 2017; NIH, 2018). When applied properly, it can play a central role in the drug discovery pipeline (Wyatt et al., 2011; Field et al., 2017). Besides VL and CL specificities, additional criteria are considered for TPP establishment, for instance, drug candidates should present broad-spectrum activity, in terms of distinct species (and strains) and geographic regions (DNDi, 2018a, 2018b).

Because the classical pipeline of *de novo* drug discovery is a high cost and time-consuming approach (Fig. 1), *short-term* strategies have been considered as a promising answer to accelerate the process of novel candidates' identification and optimization (Pink et al., 2005; Charlton et al., 2018). Drug combination has been explored in leishmaniasis and trypanosomiasis treatment/clinical trials in order to increase drug efficacy, shorten the course of treatment and potentially decrease toxicity. Improvement in tolerability should be expected as two drugs can be administered below their individual dose limits, possibly reducing their side effects. Furthermore, there is a potential for combination therapy to reduce resistance in pathogenic organisms (MacLean et al., 2010; Alirol et al., 2013). It has been shown that nifurtimox-eflornithine combination therapy (NECT) can be safely used as first-line treatment for the second-stage of Human African Trypanosomiasis caused by *Trypanosoma brucei gambiense* (Priotto et al., 2009; Yun et al., 2010). A very recent study utilising a multicentre randomized clinical trial conducted in Uganda showed that NECT schemes are shorter and less expensive than eflornithine monotherapy. It is worth pointing out that this clinical trial was funded from a partnership between UNICEF/UNDP/World Bank/WHO, the Drugs for Neglected Diseases *Initiative* (DND*i*) and the Government of Uganda (Kansiime et al., 2018). VL’ clinical studies using Amphotericin B in combination with miltefosine or paromomycin have also shown promising results: both combinations were well tolerated and safe, with cure ratios that exceeded 94%. In that case, financial support was provided by a collaborative network including private foundations (Bill & Melinda Gates Foundation, the Buck Foundation, and Fondation de bienfaisance du groupe Pictet), government agencies (Swiss Agency for Development and Cooperation, Department for International Development (UK), and Spanish Agency for International Development Cooperation), and the medical humanitarian organization Médecins Sans Frontières (Rahman et al., 2017).

Drug repurposing also represents a valuable contribution in this context (Sundar and Olliaro, 2007; Pinazo et al., 2010). Since it is based on the application of approved drugs to new clinical use, this alternative potentially leads to time and cost saving schemes. Additionally, information regarding clinical safety, pharmacokinetics, pharmacodynamics and potential biological targets might be easily found and assessed in the literature (Andrews et al., 2014). Charlton and colleagues, in a recent review, addressed the importance of redirecting drugs for leishmaniasis. Several examples of repurposed drugs towards VL and CL experimental models are discussed, including antifungals, antivirals and anticancer drugs. Highly favourable is the aspect related to shortening the steps necessary for the development of a drug, since it is already available in the market in most cases (Charlton et al., 2018). In fact, successful examples can be observed in the context of the TriTryp diseases as several commercial drugs in use were indeed repurposed, such as antibiotics (paromomycin), antifungals (Amphotericin B) and anti-cancer agents (eflornithine, miltefosine and nifurtimox).

## 4 Bottlenecks in drug discovery against *Leishmania*

### 4.1 Non-scientific challenges

Primarily, drug discovery and development system demands high investment for human and financial sources and, because TriTryp diseases do not represent a substantial profitable market, pharmaceutical industry has presented minor interest in this field (Trouiller et al., 2002; Pedrique et al., 2013). Findings published in *Lancet Global Health* have reported that only 4% of 850 new therapeutic products licensed from 2000 to 2011 were exclusively targeted for neglected diseases, including: 25 new indications/formulation, 8 vaccines or other biological products (e.g. immunoglobulins) and 4 new chemical entities. Additionally, the development of new chemical entities was focused on malaria and diarrhoeal diseases (cryptosporidiosis and giardiasis). During the period of study, no new compound was registered targeting a neglected disease. Regarding leishmaniasis, two new alternatives were approved for treatment, miltefosine and paromomycin, both repurposed drugs. For Chagas disease, only a new formulation of benznidazole designed for paediatric use was developed, despite recent clinical studies that aimed to evaluate the repositioning of pozaconazole for Chagas' disease (Box 1). One combination (nifurtimox+eflornithine) was approved for sleeping sickness treatment during the same period. Clinical trials registered in WHO and NIH databases showed that the context does not tend to change in the near future: from 148,445 therapies in development, approximately 1% is for neglected diseases, from which only 23% has been performed in pharma/biotech industry (Pedrique et al., 2013).

BOX 1 **“From bench to bedside”: A lesson to be learned from Trypanosoma cruzi**

In recent years, posaconazole, a broad-spectrum second-generation triazole with antifungal activity, has emerged as a possible drug candidate to Chagas disease treatment. However, anti-*T. cruzi* activity of posaconazole in animal models failed to predict drug effectiveness in humans as treatment failure during follow up with the antifungal was higher than benznidazole (Molina et al., 2014; Morillo et al., 2017). Even the combination of posaconazole and benznidazole did not lead to improved results when compared with benznidazole monotherapy (Morillo et al., 2017). One possible explanation for the distinct results in the murine model and human trials is that the former represents the early stage of chronic Chagas disease in which the response of the drugs can be overestimated. Besides that, it has been suggested that in the late chronic stage of the disease, *T. cruzi* may have amastigote forms that could be more resistant to ergosterol inhibitors (Molina et al., 2014). As a result, although treatment can lead to severe side effects, benznidazole is still the drug of choice. One lesson to be learned is the importance of considering distinct parasitic stages, which can remain in the tissue and may show variable drug sensitivity when compared to other life cycle stages (Bern, 2017). In this sense, screening protocols designed to find molecules targeting amastigotes’ nests, for example, should be incorporated in more accurate *in vivo* models, such as bioluminescence imaging (Lewis et al., 2014), which have been used in drug discovery pipelines (Francisco et al., 2015) to allow infection visualization and drug effect instantly.”

Alt-text: BOX 1

However, the involvement of pharmaceutical companies in the neglected disease field is expanding specially when considering aspects such as drugs provision. Hotez and colleagues have compiled data from WHO showing that billions of tablets for neglected tropical diseases (NTDs) are donated by GlaxoSmithKline, Johnson & Johnson, Merck KGaA, MedPharm, Merck & Co and Pfizer, in addition to direct procurement (Hotez et al., 2009). It is essential to recognize that there are not enough investments yet from the pharmaceutical industries for leishmaniasis, and much of the cost and availability of medication is negotiated through non-governmental organizations and the WHO (den Boer et al., 2011). Specifically, WHO has been committed to work with the public and private sectors, international agencies, and non-governmental development organizations in order to guarantee access to high-quality medicines free of charge for millions of people (WHO, 2018b). Moreover, new projects have been established given the partnership of large pharmaceutical companies (e.g. GlaxoSmithKline and Novartis) with institutions such as DND*i*, Wellcome Trust and academia, especially aimed at characterizing new chemical entities with leishmanicidal and trypanosomicidal activity. As result, millions of compounds have been screened in drug discovery campaigns against TriTryps parasites by partnerships with GlaxoSmithKline and Novartis (Khare et al., 2016; Peña et al., 2015). GSK Tres Cantos, for example, has also integrated a collaborative research network for more than a decade with the Drug Discovery Unit (DDU, University of Dundee) and Wellcome Trust to discover new candidate drugs for VL and Chagas disease (Drug Discovery Unit, University of Dundee, 2018). Willie and collaborators have recently reported a potential drug candidate (DDD853651/GSK3186899) that showed *in vitro* potency and *in vivo* efficacy with appropriate pharmacokinetic, physicochemical and safety properties, justifying its continuation for human clinical trials (Wyllie et al., 2018).

**Fig. 2:**
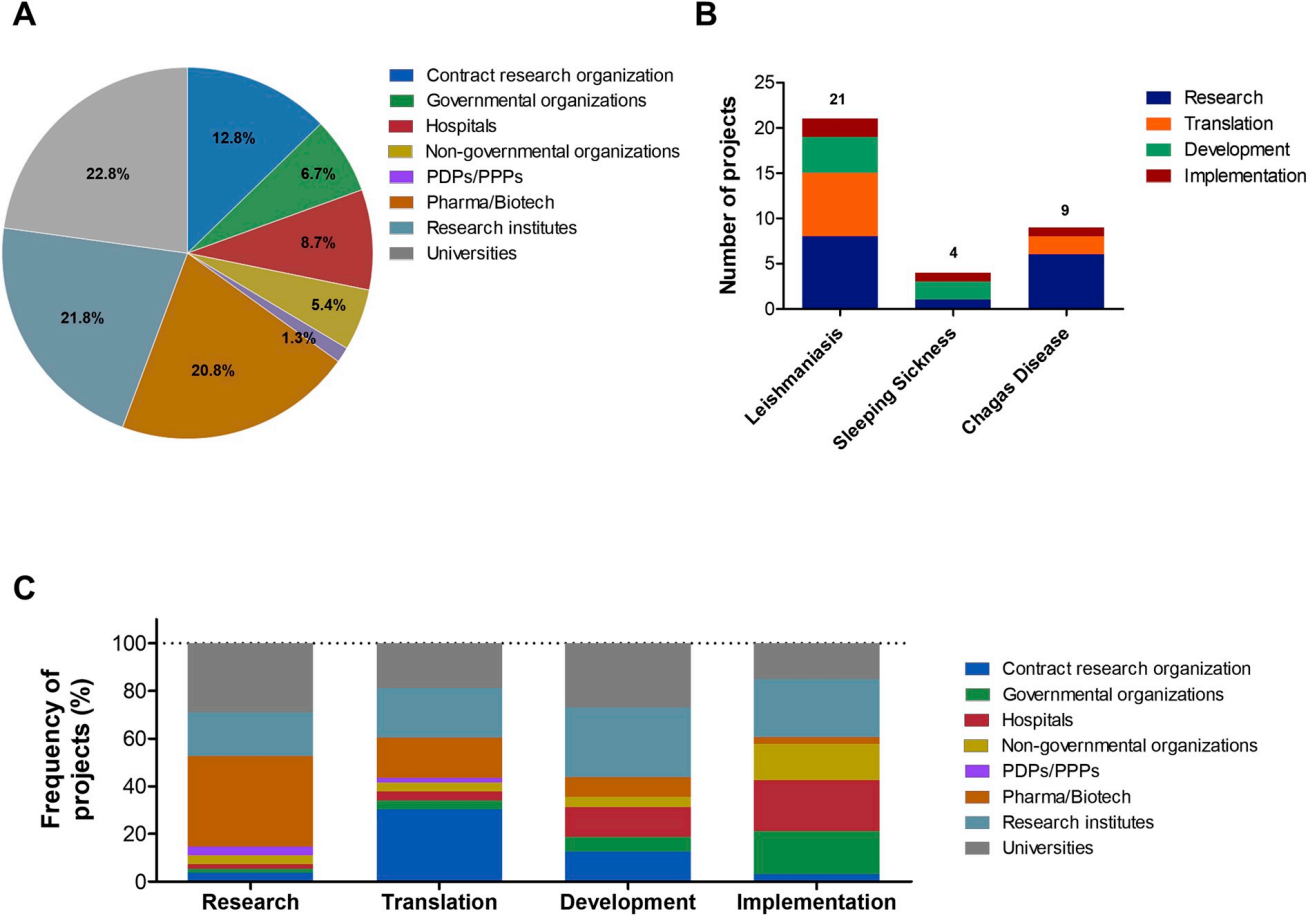
**Profile of DNDi partners. (A)** Graph illustrating general distribution of DND*i* partners. **(B)** Distribution of projects by TriTryp diseases. **(C)** Distribution of partners by each stage of drug discovery pipeline. Pie and bar charts were generated based on data available on DND*i* website (www.dndi.org) in December, 2017. Legend: Pharma/Biotech: pharmaceutical and biotechnological companies; PDPs/PPPs: product development partnership and public – private partnership, respectively; NGOs/IOs: non-governmental organizations and international organizations, respectively; Contract Research Organizations: organizations providing support to the pharmaceutical and biotechnology industries.

Drugs for Neglected Diseases *initiative* (DND*i*) is a very well succeeded example of a non-profit organization that drives efforts to identify and fill the gaps across the drug discovery and development pipelines (Table 2). Today, DND*i* has more than 150 partners (Fig. 2A), such as universities, research institutes, pharma/biotech companies, ministries of health and governmental organizations, working directly in the development of drugs against TriTryps. Thirty-four projects are in development at different stages (Fig. 2B), being the majority focused on leishmaniasis (21 projects), followed by Chagas disease (9 projects) and sleeping sickness (4 projects). The activities’ portfolio currently consists on:

(i) research: library screenings and lead compounds optimization;
(ii) translation: test of fexinidazole and new benznidazole regimens for the treatment of chronic Chagas' disease and evaluation of nitroimidazoles and oxaborole compounds as well as combination therapy for leishmaniasis;
(iii) development: new treatment regimens for HIV/VL infected patients and combinatory therapy with amphotericin B and miltefosine for post-kala-azar dermal leishmaniasis as well as the assessment of fexinidazole and acoziborole against *T. brucei*;
(iv) implementation: access to combinatory therapy of eflornithine and oral nifurtimox for human African trypanosomiasis treatment, new benznidazole formulation for children with Chagas disease and sodium stibogluconate+paromomycin scheme for leishmaniasis in East Africa.

**Table 2:** List of compounds on current DNDi pipeline.

Compound	Target disease	Phase
Aminopyrazoles	VL/CL	Research Phase (Lead Optimization)
CGH VL Series 1	VL	Research Phase (Lead Optimization)
DNDI-5421 and DNDI-5610 Oxaboroles	VL/CL	Research Phase (Lead Optimization)
Leish H2L	VL	Research Phase (Lead Optimization)
CpG D35	PKDL/CL	Translation Phase (Pre-clinical)
DNDI-6148 Oxaborole	VL/CL	Translation Phase (Pre-clinical)
DNDI-0690 Nitromidazole	VL/CL	Translation Phase (Pre-clinical)
DNDI-5561	VL	Translation Phase (Pre-clinical)

PKDL: Post-kala-azar dermal leishmaniasis.

Data available at www.dndi.org. Aug, 2018.

The partners’ participation outline varies in the drug discovery pipeline (Fig. 2C), in which pharma/biotech companies mainly contribute in the first steps of the process (especially in research) whereas governmental and non-governmental organizations play their role mostly in advanced stages, such as implementation. Universities, research institutes and Contract Research Organizations are key players in the whole process (DNDi, 2018c).

In this sense, it is worth-mentioning that governmental agencies are also investing strengths to encourage drug discovery/development initiatives related to TriTryp diseases. NTD Priority Review Voucher (PRV), for example, was implemented in the United States in 2007 as a powerful resource to expedite the development of new therapies or biological products to prevent certain neglected diseases. As so, miltefosine in 2014 has been granted accelerated approval (FDA, 2014) and in 2017 benznidazole also received a PRV for Chagas disease paediatric treatment (FDA, 2017). The Pros of this program are the release of the review result by FDA in 6 months, no cost to the U. S. taxpayer, no effect on FDA review of other compounds and turning these therapies accessible to U.S. residents. The Cons are related to voucher eligibility, the high cost of neglected diseases clinical trials and return on the investment, which may be not profitable when the voucher is given to a not novel product (Berman and Radhakrishna, 2017; Ridley, 2017). Nevertheless, more NTDs were added to the voucher eligible list in 2014/15 (Berman and Radhakrishna, 2017).

Still, unlike the profitable disease drug discovery and development that involves multicentre coordination and high investment, a relevant portion of drug prospection for neglected diseases relies on academic and research institutions. This process may indicate a fragmented and non-integrated approach, resulting in few potential compounds that indeed follow up into a more advanced product. An additional disrupting factor is the lack of central database capable of concentrating positive and negative results from different research groups. As a consequence, for example, compound libraries are redundantly screened in the same models, leading to unnecessary losses and time wasting (Pécoul, 2004; Goupil and McKerrow, 2014). Even so, efforts to circumvent these obstacles have been made by the provision of publically available datasets and chemical libraries. Medicines for Malaria (MMV) Pathogen Box, for example, has been modeled help speed up neglected disease drug discovery. This project supplies drug-like molecules active against neglected diseases of interest free of charge to any research laboratory in the world. In return, researchers are asked to share any data generated in the public domain within 2 years. The scientific articles generated from this partnership become available through Pathogen Box website (MMV Malaria Box, 2018; Pathogen Box, 2018). Another example to be highlighted is the ChEMBL, launched in 2009. Basically it comprises a manually curated chemical database of bioactive molecules that provides several parameters of biological activity, such as EC_50_, Kd, and Ki.Compiled data can be analyzed to develop compound screening libraries (Brenk et al., 2008; EMBL-EBI, 2018). These examples of initiatives have the potential to build global bridges between scientists accelerating the research in the field of neglected diseases.

These bottlenecks have been recently overcome with several advances achieved due to Public – Private – Partnerships (PPP) initiatives, combining academic knowledge and pharma/biotech expertise in an efficient *modus operandi* network. In this context, academia is responsible for activities such as the development of new *in vitro* and *in vivo* models, targeting identification and validation, and the improvement of screening tools, whereas industry provides chemical and natural product libraries, infrastructure, technical know-how, large-scale manufacturing, and financial support (Gustavsen and Hanson, 2009; Chatelain and Ioset, 2011). Initiatives to support proposals for some diseases, including VL, in Hit-to-Lead and Product Development Platforms, for example, are one of the Global Health Innovative Technology (GHIT) Fund's goals (GHIT fund, 2018). The major purpose, in this case, is to fuel the creation of a connection between early drug discovery and product development platform that begins with the lead-optimization step. The GHIT Fund routinely announces investment opportunities for the Target Research Platform (TRP) in partnership with the Wellcome Trust. Possible collaborations are encouraged by including co-working with Medicines for Malaria Venture (MMV), Drugs for Neglected Diseases Initiative (DNDi), and the Global Alliance for TB Drug Development (GATB).

### 4.2 Scientific challenges

In the last decade, technology advances have allowed the establishment of high throughput screening against *Leishmania* parasites, meaning that millions of compounds have been tested in an attempt to identify new antileishmanial agents (Peña et al., 2015; Khare et al., 2016). Nonetheless, leishmaniasis treatment still remains limited and no compounds were recently developed or registered, which highlights that there are some key knowledge gaps in the *Leishmania* drug discovery pipeline that need to be addressed, such as the lack of systematic studies (e.g., strains panel and “time-to-kill” assay) that demonstrate hit potential and optimization for clinical use (hit-to-lead process) and limited pharmacokinetic and pharmacodynamics (PK/PD) studies.

Normally, *in vitro* cell-based assays are the starting points in the drug discovery pipeline (Fig. 1). Target-based approach is less applied in *Leishmania* drug discovery, due to the limited number of fully validated targets and issues of confirming on-target effects of active compounds (Reguera et al., 2014). Table 3 exemplifies some high throughput screening (HTS) campaigns focusing on *Leishmania* proteins and related limitations. The only antileishmanial drug with defined target is Amphotericin B, which binds preferentially to parasite's ergosterol (Saha et al., 1986).

**Table 3:** Examples of *Leishmania* targets used in HTS campaigns.

Protein	Abbreviation	Target limitations	Reference
Casein kinase 1.2	LmCK1.2	High degree of conservation between the parasite and human CK1 isoforms represents a challenge for the identification of parasite-specific CK1 inhibitors with limited side effects on host kinases (Rachidi et al., 2014).	(Durieu et al., 2016)
N-myristoyltransferase	NMT	Very few proteins have been experimentally validated as N-myristoylated in *Leishmania* species, impairing the understanding of its essentiality in parasite biology (Tate et al., 2014)	(Bell et al., 2012)
Cdc2-related kinase 3	CRK3	Poor correlation between potency against the target and anti-parasitic activity, suggesting some unknown aspect of CRK3 biology in *Leishmania* or unknown bioavailability within the parasitophorous vacuole (Jones et al., 2018)	(Walker et al., 2011)
Pteridine reductase	PTR1	Despite extensive work, no inhibitors for this target have been progressed to preclinical development (Field et al., 2017)	(Cavazzuti et al., 2008)

***In vitro studies.*** The development of new therapeutics focuses on screening potentially effective compounds in parasite growth/multiplication assays. There are several screening assays available against *Leishmania* based on different stages of the parasites: promastigotes (Siqueira-Neto et al., 2010), axenic amastigotes (Nühs et al., 2015) or intracellular amastigotes (Siqueira-Neto et al., 2012). Screenings for axenic forms of *Leishmania* present several advantages; (i) limited number of parasites is sufficient to test many compounds; (ii) faster read-outs; (iii) higher throughput; and (iv) reproducibility.

These advantages become particularly important during the initial screening of large sample libraries. However, it is worth noting that axenic form-based assays may present a significant caveat: substantial metabolic differences between the amastigote and promastigote stages may lead to selection of misleading candidates (i.e. selection of molecules active specifically against promastigote forms, which are irrelevant for disease progression and treatment) (Croft et al., 2006b; Siqueira-Neto et al., 2012). Still, although axenic amastigotes screenings are performed with the clinically relevant parasite stage, the difficulty in obtaining axenic amastigotes for several *Leishmania* species may slow down more comprehensive *in vitro* studies. Not only methodological obstacles are recognized, but biological disadvantages can explain some limitations of this type of assay, such as the fact that drug penetration in the host cell is not evaluated, neither is the activity in the phagolysosomal environment (acidic milieu) and the lack of correlation between selected compounds in axenic forms screenings and intracellular amastigote assays. Siqueira-Neto et al. showed that 50% of the hits chosen against the intracellular amastigote are not selected in the promastigote screening (Siqueira-Neto et al., 2012), and De Rycker et al. described a high false-positive rate for the axenic amastigote assay (De Rycker et al., 2013).

In 1986, S. Croft defined a number of requirements for an ideal *in vitro* assay: amastigotes as target, a dividing population, quantifiable and reproducible measures of drug activity and standard drugs activity in concentrations achievable in serum/tissues (Croft, 1986). The intracellular amastigote assay usually involves primary isolated macrophages as host cells (mouse peritoneal macrophages, mouse bone-marrow-derived macrophages or human blood monocyte-derived macrophages) or human-monocyte transformed macrophages (THP-1, U937, and HL-60). In differentiated non-dividing macrophages, drug activity can be assessed in a realistic way as it is possible to control the multiplicity of infection throughout infection time points. The activity of tested drugs is measured by microscopic counting of infected cells and number of amastigotes per macrophage or by colorimetric/fluorimetric methods (Croft et al., 2006b). In addition, the criteria established by the GHIT Fund represent an interesting set of standards needed for selection of 'hit compounds' for leishmaniasis chemotherapy:

i. a given hit should present a 50% effective concentration (EC_50_ value) lower than 10 μM against intracellular amastigotes of *Leishmania* sp.,
ii ii. for the *in vivo* model of VL (i.e. mouse or hamster infected with *L. infantum* or *L. donovani*), treatment schemes should result in 70% reduction of liver parasite load after up to 5 doses of 50 mg/kg orally one or two times a day.

Perhaps, when all these criteria are met, the comparison of data between different research groups will be more precise, facilitating the advance of the effective selection of active compounds.

High content assay combined with high throughput screening (HCS/HTS), and automated image analysis, has been highlighted as it combines the efficiency of HTS with multiparameter readout, providing phenotypic information in the whole cell (Siqueira-Neto et al., 2012). This setup enables qualitative and quantitative systematic evaluations of various cellular phenomena (for example, absence or reduction of parasites in host cells), being used to measure compound activity. All potential targets in this case will be exposed to the tested compounds. In comparison to traditional assays that provide information mainly on parasite viability, the use of HCS technologies also allows the assessment of potential toxicity against the host cells and to observe morphological changes that can provide useful information to understand the mode of action of the compounds of interest (Zanella et al., 2010).

An alternative methodology developed to screen compounds against *Leishmania* is the use of genetically modified parasites expressing reporter genes, such as green fluorescent protein (GFP) or luciferase. Many recombinant parasites carrying a reporter gene either as an episomal copy or genome-integrated are currently available (Singh and Dube, 2004; Lang et al., 2005). Despite the advantages of engineered parasites in the development and improvement of biological assays, genetic modifications can lead to modifications in parasite metabolism and loss of virulence (Rocha et al., 2013).

The amastigote-infected macrophage assay is undoubtedly the gold standard for *Leishmania* drug discovery. The main drawback is the low hit-rate, partially explained by slow replication of amastigotes (Tegazzini et al., 2016), making the cytocidal and cytostatic effect of candidates even more challenging to determine. Khare et al., in a collaborative effort led by The Genomics Institute of the Novartis Research Foundation (GNF) supported by the Wellcome Trust and in partnership with several Universities, tested 3 million compounds against *L. donovani* axenic amastigotes. Also, the activity against *T. cruzi* intracellular amastigotes and *T. brucei* bloodstream trypomastigotes was evaluated. GNF5343 was identified as a hit against *L. donovani* and *T. brucei*. Although GNF5343 has not been identified in the *T. cruzi* screening, its potent anti-*T. cruzi* activity was assessed as well. The optimization of GNF5343, focused on improving bioavailability and potency while inhibiting *L. donovani* growth within macrophages led to GNF6702, which cleared parasites for each of the *in vivo* infection models tested (Khare et al., 2016). Therefore, new compounds can be identified from axenic model assays, and then associated with intracellular amastigotes assays for further characterization. It is relevant to keep in mind that if a certain compound is active in an axenic amastigote model but is incapable of eliminating the parasites in the intracellular model, this could be possibly explained by the interference of host cells factors (i.e., mammalian cell plasma membrane, vacuolar membrane, vacuolar pH). Yet, these candidates can be considered ‘starting point molecules’ with potential for chemical optimization aiming to circumvent cells obstacles.

Several systems with different strains/species are employed in primary screenings of antileishmanial compounds, making it difficult to compare data from distinct laboratories. Moreover, it has been demonstrated that the activity of some antileishmanial drugs is host cell dependent (Seifert et al., 2010).

It is also important to include recent isolates of *Leishmania* species/strains from the field for *in vitro* as well as in *in vivo* tests, avoiding activity of hits against laboratory-adapted parasites. Although it is known that reference organisms isolated present consistency and uniformity, *Leishmania* virulence fluctuates over time after several *in vitro* passages (Miguel et al., 2011; Zauli-Nascimento et al., 2010). Additionally, it is essential to establish secondary assays that could facilitate the *in vitro*/*in vivo* translation, providing the basis towards the construction of a solid go-no go decision matrix for leishmaniasis drug discovery. For example, *in vitro* evaluation of antileishmanial drug activity has been limited by determining EC_50_ values at specific timepoints. Only recently, assessment of the minimal exposure time required to exert full leishmanicidal activity, also known as “time-to-kill” assay, was reported (Maes et al., 2017; Voak et al., 2018) and should be considered a trend for future *in vitro* assays. Unraveling the relation of the time-to-kill assay to drug resistance and treatment outcome can be a powerful tool to prioritize selected drug candidates.

***In vivo studies.*** The use of animal models is still necessary to establish candidate anti-protozoan activity as well as pharmacokinetics properties (absorption, distribution, metabolism, excretion - ADME) and safety profile. There are many animal models used for anti-*Leishmania* candidates’ tests, but their predictive validity is often low due to incomplete translation to the human disease. In this scenario, it is extremely relevant to determine the most effective animal model for drug discovery for each species, especially considering critical aspects as PK/PD and drug efficacy in these models (Freitas-Junior et al., 2012; Mears et al., 2015).

For VL, the most suitable models are: (i) BALB/c mice and Syrian golden hamsters (primary tests); (ii) dogs (secondary tests) and (iii) monkeys viz., squirrel, Vervet and Indian languor monkeys as tertiary tests. Hamsters are recognized as a relevant model for VL caused by *L. donovani*, as they mimic features of the human disease showing progressive increase in parasite burden, cachexia, hepatosplenomegaly, pancytopenia, hypergammaglobulinaemia and ultimately death (Garg and Dube, 2006). PKDL is a complication associated with VL caused by *L. donovani* and for which there is no animal model of infection established. Regarding CL, there are several species causing different clinical manifestations, which bring complexity to the establishment and validation of models with features similar to humans with respect to etiology, pathophysiology, symptomatology and response to the therapeutic or prophylactic agents. There is no validated animal model for CL. Mears et al. revised the current animal models and suggested the as most suitable options for CL drug discovery: *L. major*–C57BL/6 mice (or –Vervet monkey, or –Rhesus monkey), *L. tropica*–CsS-16 mice, *L. amazonensis*–CBA mice, *L. braziliensis*–golden hamster (or –Rhesus monkey) (Mears et al., 2015).

Latest application of real-time *in vivo* imaging technology has enabled faster and more accurate analyzes of measurable signals associated with cells in living organisms. Basically, animals are infected with bioluminescent or fluorescent transgenic *Leishmania* for subsequent parasite burden quantification by fluorescence intensity (RFU) or photons. Several successful models are reported for both VL and CL (Table 4). This approach provides substantial advantages over currently available animal model systems for *in vivo* study as more sensitive image-based technology may improve low parasite burden detection and the ability to acquire real-time data on progression and spread of the infection. The classical methods of *in vivo* parasite load determination require animal euthanasia at various times points after infection, making the process laborious, time consuming, and unviable for automation (Okuno et al., 2003). Also, recovering parasites from infected tissues and organs can be affected by bacterial and/or fungal contamination; especially after extended periods of amastigote to promastigote differentiation that are required for parasite quantitation by widely used protocols (i.e., limiting dilution assay).

**Table 4:** Real-time *in vivo* imaging models for *Leishmania.*

Disease	Species	Animal	Gene reporter	Reference
VL	*L. donovani* Ld1S/MHOM/SD/00-strain 1S	BALB/c mice	luciferase	(Melo et al., 2017)
VL	*L. donovani* MHOM/ET/67/HU3	Syrian golden hamsters	luciferase	(Rouault et al., 2017)
VL	*L. infantum* MHOM/FR/94/LPN101	BALB/c mice	luciferase	(Cannet et al., 2016)
VL	*L. infantum chagasi* MHOM/BR/1972/LD	Syrian golden hamsters	luciferase	(Reimão et al., 2015)
CL	*L. braziliensis* MHOM/BR/94/H3227	BALB/c mice	luciferase	(Coelho et al., 2016)
CL	*L. tropica* MHOM/IL/2006/LRC-L590	Sprague-Dawley rats	luciferase	(Talmi-Frank et al., 2012)
CL	*L. amazonensis* MHOM/BR/75/LTB0016	BALB/c mice	near-infrared protein (iRFP)	(Oliveira et al., 2016)

Unfortunately, most of compounds do not reach to clinical stage. A recent systematic review identified 145 published VL clinical trials, with data from ∼27k patients. Only 0.75% (203 patients) were enrolled in studies with other drugs excluding pentavalent antimonial, Amphotericin B deoxycholate, miltefosine, Amphotericin B lipid-associated formulations, paromomycin, pentamidine or sitamaquine (Bush et al., 2017). Indeed, all DND*i* clinical trials that are ongoing are with reference drugs (Table 5). However, thanks to partnerships that have been growing among different institutions, there is an important but small number of programs in DNDi's portfolio at different stages of drug discovery/development, including lead optimization and preclinical phases, as listed in Table 2 (DNDi, 2018c). These programs focus mainly on VL and CL, but it has to be emphasized that the immunomodulator CpG D35 may represent a promising drug to fight the parasitic infection responsible not only for CL, but also for PKDL.

**Table 5:** Ongoing DND*i* clinical trials.

Identifier	Country	Phase	Treatment	Goal	Project start:
NCT03129646	Ethiopia, Kenya, Sudan, Uganda	III	SSG^a^+paromomycin, paromomycin+miltefosine	Assess the efficacy and safety of two combination regimens for the treatment of primary VL patients in Eastern Africa.	January 2018
CTRI/2017/04/008421; NCT03399955	India, Bangladesh, Sudan	II	AmBisome^®^^b^, AmBisome^®^+miltefosine, paromomycin+miltefosine	Determine safety and efficacy profiles of two treatment regimens for patients with PKDL.	March 2015
NCT02687971	Colombia, Peru	II	miltefosine+thermotherapy	Further explore opportunities to better use the existing approved treatment approaches for CL when used in combination.	June 2015
CTRI/2015/05/005807	India	III	AmBisome^®^, AmBisome^®^+miltefosine,	Identify and deliver a safe and highly effective treatment for VL in HIV co-infected patients.	September 2011

Data available at www.dndi.org. Aug, 2018.

a SSG: sodium stibogluconate.

b AmBisome: liposomal Amphotericin B.

## 5 Conclusions and final considerations

TriTryp diseases are intimately linked with poverty and scarceness of resources, falling excessively on the poorest segments of the global population. Leishmaniasis is related to a number of factors, including underfeeding, human displacement, poor housing, and illiteracy. Being responsible for the ninth largest disease burden among individual infectious diseases, leishmaniasis should not be ignored in discussions of tropical disease priorities. The combination of its complex epidemiology and ecology, the lack of easily applied tools for case management and the paucity of current incidence data often result in failure of policy-makers to recognize its importance. Based on the economic and social context of this disease, advancing on novel experimental chemotherapeutic alternatives remains imperative.

Despite the recent advances observed in drug discovery for leishmaniasis, scientific and non-scientific bottlenecks need to be addressed in order to push forward the development of new treatment options. Scientific issues can lead to true-positive screening hits that show poor *in vivo*/clinical translation and/or false-positive hits, possibly explained by the lack of standardized screening methods, which must consider the biological diversity not only among strains and species but also between parasite stages. There are several *in vitro* assays and different protocols that make data comparison difficult among reports/research groups. Possibly, by identifying the most appropriate biological model combined with available and suitable tools will lead to assay standardization. In terms of leishmanicidal activity of a given candidate, the ability of the leading compound to permeate the host cell and be active in an acidic environment has to be taken into consideration in screening against intracellular amastigotes as well. Further, immunomodulation of the host cell could represent a good target for drug development against the disease. Another Achilles' heel on drug development is the limited PK/PD studies and clinical evidence to validate preclinical research. In terms of validation, an interesting alternative would be the exchange of tests between different laboratories that have the expertise in this type of analysis.

From a non-scientific point of view, the slight interest from pharmaceuticals companies together with the fact that not all academic and research institutions may have integrated approaches in the drug discovery pipeline due to (funding/expertise) restrictions also contribute to hamper drug discovery. Though, the involvement of the abovementioned institutions has grown and shown encouraging results. Forging successful global partnerships between private and public sector will be fundamental to integrate scientific findings into the continuum of care and translating science from bench to bedside. Furthermore, the role of organizations such as DNDi must be significantly valued as they have the potential to serve as platforms for advancing the implementation of public health policies towards the development of novel effective drugs. Besides, DNDi is involved in PPP's and has been playing a central role in the discovery of new compounds against NTD's with a portfolio of molecules/new formulations directed to all stages of the drug discovery pipeline (research, translation, development and implementation).

Dealing with diseases of such relevance worldwide must require the incorporation of an interdisciplinary and cross-functional approach such as:

i. Government and private funding for basic and clinical research projects through direct investments and incentives to both academia and the private sector;
ii. Investment in health surveillance and public outreach program;
iii. Public awareness policies, so as to involve not only social participation but also non-governmental organizations.

Several examples of collaborative network initiatives have been shown to positively influence the resolution of complex issues associated with the discovery of new therapies not only directed to leishmaniasis but also to other neglected diseases. The support of governments, foundations, non-profit organizations, academia and industry should be seen as a necessary ally for the development of new technologies public health policies towards the control of such devastating diseases.

## Conflicts of interest

The authors declare no conflict of interest.

## Funding

LMA received a doctorate scholarship from CNPq (gs1:Conselho Nacional de Desenvolvimento Científico e Tecnológico, #140907/2013-0) and TCSF was awarded a FAPESP (gs2:Fundação de Amparo à Pesquisa do Estado de São Paulo) doctoral fellowship (#15/10436-6). FRG is supported by grants from FAPESP (#15/24595-9) and CNPq (#309764/2015-7). DCM is supported by a FAPESP Young Investigator Award (#2014/21129-4).

## Appendix A Supplementary data

Data Profile Data Profile
